# Salivary Ferritin Changes in Patients with COVID-19

**DOI:** 10.3390/ijerph19010041

**Published:** 2021-12-21

**Authors:** Lorena Franco-Martínez, José J. Cerón, María R. Vicente-Romero, Enrique Bernal, Alberto Torres Cantero, Fernando Tecles, Cristina Sánchez Resalt, Mónica Martínez, Asta Tvarijonaviciute, Silvia Martínez-Subiela

**Affiliations:** 1 Interdisciplinary Laboratory of Clinical Analysis, Interlab-UMU, Regional Campus of International Excellence ‘Campus Mare Nostrum’, University of Murcia, 30100 Murcia, Spain; jjceron@um.es (J.J.C.); ftecles@um.es (F.T.); cristinasr@um.es (C.S.R.); silviams@um.es (S.M.-S.); 2 Unit of Microbiology, Hospital General Universitario Reina Sofía, Universidad de Murcia, 30003 Murcia, Spain; mrvr79@gmail.com; 3 Unit of Infectious Diseases, Hospital General Universitario Reina Sofía, Universidad de Murcia, 30003 Murcia, Spain; ebm.hgurs@gmail.com (E.B.); monica.martinez.3m@gmail.com (M.M.); 4 Preventive Medicine, Hospital Clínico Universitario Virgen de la Arrixaca, IMIB, Universidad de Murcia, 30120 Murcia, Spain; alberto.torrescantero@gmail.com

**Keywords:** COVID-19, ferritin, non-invasive sample, biomarker

## Abstract

High ferritin serum levels can be found in patients with macrophage activation syndrome, and increased serum ferritin due to cytokine storm have been reported in severe COVID-19 patients. Saliva is being increasingly used in COVID-19 tests as a diagnostic sample for virus detection and quantification. This study aimed to evaluate the possible changes in ferritin in saliva in COVID-19 patients. In addition, the effects of different inactivation SARS-CoV-2 treatments in ferritin measurements in saliva, the correlation between ferritin in saliva and serum, and the possible effects of correction of ferritin values by total protein were assessed. Ferritin was measured in saliva from healthy (*n* = 30) and COVID-19 (*n* = 65) patients with severe, (*n* = 18) or mild (*n* = 47) disease, depending on the need for nasal flow oxygen or assisted respiration. Ferritin was also measured in paired serum and saliva samples (*n* = 32) from healthy and COVID-19 patients. The evaluated inactivation protocols did not affect the assay’s results except the addition of 0.5% SDS. Significantly higher ferritin was found in the saliva of COVID-19 patients (median; 25–75th percentile) (27.75; 9.77–52.2 µg/L), compared with healthy controls (4.21; 2.6–8.08 µg/L). Individuals with severe COVID-19 showed higher ferritin values in saliva (48.7; 18.7–53.9) than mild ones (15.5; 5.28–41.3 µg/L). Significant correlation (r = 0.425; *p* < 0.001) was found between serum and saliva in ferritin. Ferritin levels were higher in COVID-19 patients in serum and saliva, and the highest values were found in those patients presenting severe symptomatology. In conclusion, ferritin in saliva has the potential to be a biomarker to evaluate severity in patients with COVID-19.

## 1. Introduction

COVID-19 is an infectious disease caused by a novel enveloped RNA virus (SARS-CoV-2), resulting in severe pneumonia [1] that first emerged in December 2019 in Wuhan (Hubei, China). The disease could cause varying degrees of illness [1], with severity ranging from asymptomatic infection or mild disease characterized by dry cough, fever, dyspnea, and fatigue to critical illness with respiratory failure, acute respiratory distress syndrome (ARDS), and death [2]. The pathogenicity of novel SARS-CoV-2 and its effects on the immune system have not been completely understood, although some information can be found in this regard [3]. However, one of the primary causes of death in COVID-19 patients is an inflammatory cytokine storm [4], defined by the excessive and uncontrolled release of pro-inflammatory cytokines, also described in other infections caused by pathogenic coronaviruses [5]. Therefore, the study of inflammatory markers is considered of importance in this disease [6].

Serum ferritin is an iron storage protein with a key role in regulating cellular oxygen metabolism, and its measurement has become a valuable method to evaluate the status of intracellular iron [7]. In addition, in recent years, ferritin has been described as an essential molecule in the immune system with immunosuppressive and pro-inflammatory effects, and several studies showed its role as an acute-phase protein in an array of pathologies [8,9,10,11,12]. In this way, elevated serum ferritin levels may suggest the presence of an iron overload state, but it is also a marker of inflammatory, autoimmune, infectious, or malignant status [9,13,14,15,16,17]; therefore, is a sensitive but unspecific biomarker. Extremely high ferritin levels can be found in patients with macrophage activation syndrome [18] that is a clinical condition very similar to the severe COVID-19 patients [2]. In addition, increased levels of ferritin due to cytokine storm have been reported in severe COVID-19 patients [19,20,21], showing those severe patients higher serum ferritin in comparison with moderate cases [17].

Saliva is a fluid of high potential use in medical sciences [22]. In particular, in relation to COVID-19, it can be used in its diagnosis and for monitoring the antibody response [23]. In addition, analytes related to the immune response, such as adenosine deaminase, have been shown to change in the saliva of COVID-19 patients [24]. However, there are only a few studies that measure ferritin in saliva and, to the best of the authors’ knowledge, none in COVID-19 patients. Thus, the objective of this study is to evaluate the possible changes in ferritin levels in the saliva of COVID-19 patients. In addition, the effects of different sample treatments in ferritin levels in saliva, the correlation between ferritin in saliva and serum, and the possible effects of correction of ferritin values by total protein were evaluated.

## 2. Material and Methods

### 2.1. Participant and Samples

In all cases, participants were asked not to eat, drink, smoke, or brush their teeth for at least 1 h before saliva collection. None of the participants showed any apparent evidence of oral or other systemic diseases. Saliva was collected under supervision by passive drool using 5 mL polystyrene tubes with round bottoms [25]. Samples with blood contamination (determined by visual inspection) were excluded from the study. Immediately after collection, the saliva was stored on ice until arrival at the laboratory, centrifuged (4500 rpm, 10 min, 4 °C), and supernatants were stored in Eppendorf tubes at −80 °C until further analysis.

### 2.2. Ferritin Measurement in Saliva

Ferritin was measured by a commercial immunoturbidimetric assay that uses polyclonal anti-human ferritin antibodies (Tina-quant ferritin, Roche Diagnostics, Indianapolis, United States) in an automated analyzer (Olympus AU400 automated biochemical analyzer, Olympus Diagnostica GmbH, Ennis, Ireland). The assay was adapted from serum to saliva as described previously [26].

### 2.3. Effect of Different SARS-CoV2 Inactivation Protocols in Salivary Ferritin Concentrations

A pilot experiment was designed to investigate the effects of five effective SARS-CoV-2 inactivation protocols [27,28] in salivary ferritin measurements. For this purpose, saliva samples with high and low ferritin concentrations from 5 subjects (3 women, aged between 25 and 45 years old) were selected, divided into 6 aliquots, and treated as follows: (1) no inactivation treatment (NT Group); (2) heated at 65 °C for 30 min (H65 Group); (3) heated at 92 °C for 15 min (H92 Group); (4) the addition of (10% *w*/*v*) sodium dodecyl sulfate (SDS) to a final 0.5% concentration and 30 min incubation at room temperature (SDS Group); (5) the addition of (10% *v*/*v*) NP-40 to a final 0.5% concentration and 30 min incubation at room temperature (NP Group); (6) the addition of (10% *v*/*v*) Triton X-100 to a final 0.5% concentration and 30 min incubation at room temperature (TX100 group). Ferritin concentrations were measured in the same run as described above.

### 2.4. Salivary Ferritin Concentrations in COVID-19 Patients

Saliva samples from 95 individuals were collected and classified into two groups:

– Healthy group (HG), which included individuals who did not have any clinical sign of disease for at least 4 weeks before sampling and were negative to SARS-CoV-2 infection by RT-PCR (*n* = 30, 16 men and 14 women, aged between 23 and 75 years old).
– Diseased group (DG), which included individuals with clinical signs of two levels of severity and a confirmed COVID-19 diagnosis (*n* = 65, 34 men and 31 women, aged between 24 and 91 years old). Patients were divided according to disease severity into mild (*n* = 47, no need for oxygen supplementation or conventional oxygen therapy) and severe (*n* = 18, requiring nasal flow oxygen or assisted respiration) [29]. In all cases, samples were taken on the day of hospital admission.

The assay for SARS-CoV-2 detection involved RNA extraction and quantification by an RT-PCR with a commercial kit (FTD SARS-CoV-2, Siemens) from nasopharyngeal swabs (NPS).

For biosecurity reasons, all these samples were inactivated using 0.5% NP-40 by using the protocol described above.

### 2.5. Comparison with Serum Ferritin

Ferritin was measured in paired serum and saliva samples from HG (*n* = 12) and DG (*n* = 20) groups, and results between the two biofluids were compared. In addition, the possible correlation in ferritin levels between serum and saliva was evaluated.

### 2.6. Protein Measurement in Saliva

To evaluate the possible influence of protein content of each sample on the results, protein in saliva was measured using a colorimetric assay (protein in urine and CSF, Spinreact, Spain) adapted for its use in automatic analyzers (Olympus UA600 automated biochemical analyzer, Olympus Diagnostica GmbH, Ennis, Ireland) following the manufacturer’s instructions. Results for ferritin concentration in saliva samples were divided by its protein content.

### 2.7. Statistical Analysis

Normality in the distributions was evaluated using the D’Agostino and Pearson omnibus normality test. To evaluate the effects of the different inactivation protocols on ferritin in saliva, data were normalized considering NT as 100% and assessed using Dunn’s multiple comparisons test. Since data did not follow a parametric distribution, differences between healthy controls and COVID-19 patients were assessed by Mann–Whitney test. To determine the correlation between the two methods in both biological matrices (serum and saliva), as data did not follow a Gaussian distribution, Spearman’s correlation test was performed. The correlation was considered excellent if r ≥ 0.93; good if r was =0.80 to 0.92; fair if r = 0.59 to 0.79; poor if r was ≤ 0.59 [30].

Statistical analyses were performed with the statistical package GraphPad Prism 6 (GraphPad Software, San Diego, CA, USA), and *p* values less than 0.05 were considered statistically significant.

A post hoc power analysis was conducted using the values obtained to verify the null hypothesis. By using the mean and standard deviation of ferritin in healthy and COVID patients and a power of 80% at a 5% level of significance, the number of individuals was calculated. The data analysis was carried out using ClinCalc statistic analyzer software (https://clincalc.com/stats/samplesize.aspx (accessed on 1 December 2021) [31]. The power analysis test indicates that 34 subjects (17 for each group) were required in order to obtain a power of 80% with a 5% level of significance.

## 3. Results

### 3.1. Effect of Different SARS-CoV2 Inactivation Protocols in Salivary Ferritin Concentrations

Results of the effects of the different inactivation protocols are shown in Table 1. A significant increase in ferritin concentrations in comparison with NT was observed in SDS (median 111%, *p* < 0.05) inactivated samples. None of the other inactivation protocols produced a significant change in ferritin concentrations. 

### 3.2. Salivary Ferritin Concentrations in COVID-19 Patients

**Figure 1:**
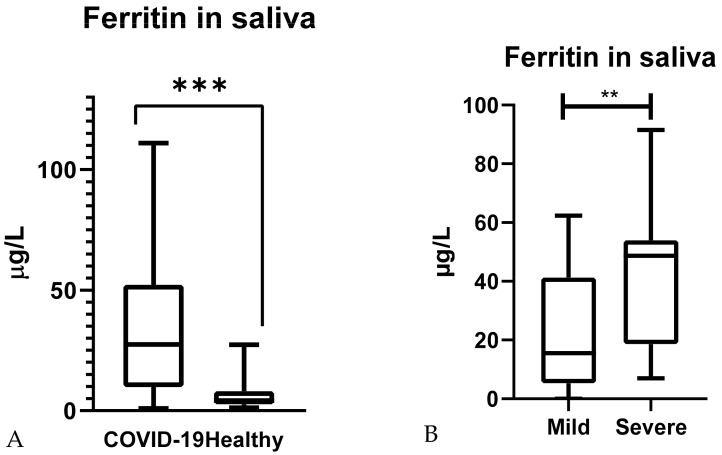
(**A**) Ferritin in saliva concentration in healthy individuals and COVID-19 patients; (**B**) ferritin in saliva in severe (required oxygen therapy) and mild (did not require oxygen therapy) COVID-19 patients. ***: *p* < 0.001; **: *p* < 0.01.

Significantly higher ferritin concentrations were found in the saliva of COVID-19 patients (median; 25–75th percentile) (27.8; 9.8–52.2 µg/L), compared with healthy controls (4.2; 2.6–8.1 µg/L) (Figure 1A). When samples from COVID-19 patients were divided into two severity groups regarding the need for oxygen support, significant differences between both groups were found (Figure 1B), with higher values in severe patients (48.7; 18.7–53.9 µg/L) in comparison with mild ones (15.5; 5.3–41.3 µg/L).

### 3.3. Comparison with Serum Ferritin

A positive correlation between ferritin in serum and saliva was observed (r = 0.425; *p* < 0.001). 

**Figure 2:**
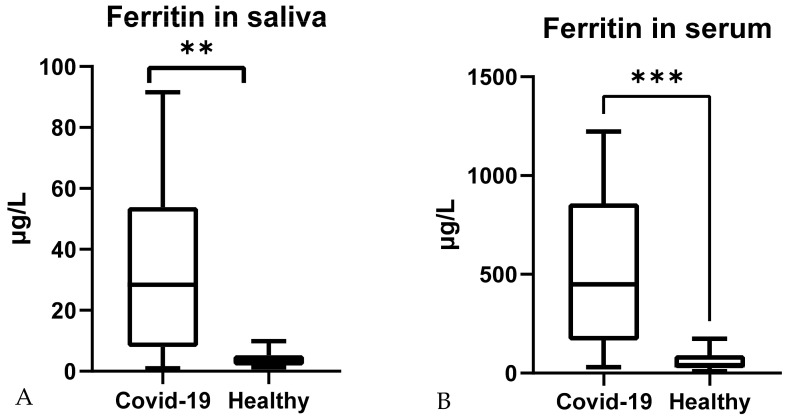
Ferritin concentrations in *n* = 32 saliva (**A**) and serum (**B**) of paired samples from healthy individuals and COVID-19 patients. **: *p* < 0.01; ***: *p* < 0.001.

Ferritin measured in paired serum and saliva samples from healthy and COVID-19 patients (Figure 2) changed significantly in both cases. Serum measurements showed lower values in healthy subjects (median; 25–75th percentile) (40.95; 25.20–90.30) µg/L than in COVID-19 patients 449.4 (165.9–859.7) µg/L (*p* < 0.001). In saliva, COVID-19 ferritin values were higher (28.35; 7.95–53.89 µg/L) than in healthy controls (3.7; 1.89–5.33) µg/L (*p* < 0.01).

### 3.4. Correction by Protein Content

The correlation between ferritin in serum and saliva corrected by protein content was positive and poor (r = 0.3; *p* = 0.017). The correlation between ferritin in saliva with and without protein content correction was strong (r = 0.85, *p* < 0.001).

When data were corrected according to their total protein content, higher ferritin values were observed in COVID-19 patients (median 0.063 µg/L mg protein) when compared with healthy controls (median 0.04 µg/L mg protein). However, the difference was not statistically relevant (*p* = 0.085).

## 4. Discussion

The present study describes the changes in ferritin levels in the saliva of COVID-19 patients for the first time. Saliva has the advantages of being a non-invasive, economic and easy to obtain sample [22,23] and, in addition, ferritin can be measured in automated analyzers, allowing its measurement in routine.

For biosecurity reasons, it is highly recommendable to inactive potentially infective samples. In this study, different inactivation protocols for the SARS-CoV-2 virus based on heat or chemicals previously reported [27,28] were evaluated in saliva, with high and low ferritin concentrations. Except for the addition of 0.5% SDS that increases ferritin measurements, the evaluated inactivation protocols did not affect the assay’s results. This contrasts with a previous study in which 65 and 92 °C heating and SDS markedly reduced adenosine deaminase concentration in saliva [24]. Therefore, if samples are expected to be inactivated for SARS-CoV-2 or other pathogens, the influence of different inactivation methods should be assessed for each biomarker. In the case of ferritin in saliva, 65 °C heating for 30 min, 92 °C heating for 15 min, or the addition of 0.5% NP-40 detergent did not affect the results significantly.

Serum ferritin values obtained in present study were similar to those previously described [6] confirming that ferritin increases in serum of COVID-19 patients. This would be in line with the increases in ferritin that occurs in infection situations [32] and other studies that showed higher ferritin serum concentration in COVID-19 patients, compared with healthy individuals [19]. 

Significant higher values of ferritin in saliva were obtained in COVID-19 patients compared with healthy controls, being the magnitude of increase in ferritin different that in serum (7.7 vs. 11.0 fold). This could be due to the fact that the correlation in ferritin between serum and saliva was modest. This weak correlation can be related to differences in ferritin kinetics in serum and saliva, since it has been described that changes in ferritin levels in saliva occur prior to changes in ferritin concentrations in blood [33]. It could be pointed out that, however, the correlation between serum and saliva in ferritin observed in this study is higher than the reported elsewhere (r = 0.27, *p* = 0.004 in volunteers with no apparent systemic disorders [34]; r = 0.064, *p* = 0.017 in patients with chronic periodontitis and type 2 diabetes mellitus [35]). Furthermore, a previous study described that ferritin in saliva, in contrast to serum, was able to discern between healthy controls and type 2 diabetes mellitus patients [35], proposing that ferritin in saliva could be more beneficial in the diagnosis of certain diseases. In our case, further studies with a larger number of individuals should be performed to evaluate which type of sample, saliva or serum, would be more sensitive to detect changes in ferritin produced in COVID-19. In addition, the study of the possible relation of ferritin in saliva with other blood or salivary biomarkers would be of high interest.

In COVID-19 patients, 3.4-fold higher ferritin in saliva was observed in patients that required oxygen supplementation in comparison to those who did not. These results are in concordance with other studies describing higher serum ferritin concentration in patients with severe manifestations [36,37,38] in comparison with less severe patients, with increases ranging from 1.5- to 5.3-fold [6]. Blood ferritin, together with other biomarkers such as C-reactive protein or D-dimer have been proposed to be used in risk stratification to predict severe and fatal COVID-19 in hospitalized patients [19,39]. This could be because serum ferritin shows major increases in severe inflammation and autoimmune response [9,13,14], processes that can be present in COVID-19 patients. In addition, several severe COVID-19 patients coursed with macrophage activation syndrome in a pro-inflammatory, prothrombotic, and hyperferritinemia syndrome [40,41].

In this study, the possible effects of correcting the results by protein content were evaluated since some biomarkers such as immunoglobulin A can be reported corrected by protein content [42,43]. When the results of ferritin in saliva were corrected according to each sample protein’s content, no improvements in correlation with serum or capacity to discern between healthy/diseased or mild/severe COVID-19 patients were found. Similar results were observed for alpha-amylase, in which the statistically significant differences between the two groups disappeared when data were corrected by protein content [25]. One possible explanation is that protein excretion in the saliva is very heterogeneous [25,44]. Therefore, the correction of ferritin values in saliva by each sample’s protein content would not be recommended, at least under the circumstances of the present study.

The present study showed some limitations. First, the number of patients could be considered low, although the post hoc power analysis conducted using the values obtained to verify the null hypothesis using the mean and standard deviation confirmed that our population was large enough to provide a power of 80% at a 5% level of significance. It would be desirable to further evaluate ferritin in saliva in a larger cohort of patients, including a more exhaustive classification of clinical manifestations and severity, response to treatment, and disease monitoring. The exploration of individual follow-up in ferritin in saliva would be also of high interest, due to the wide inter-individual range of values presented. This would allow discerning the assay’s capacity in the prognosis and follow-up of COVID-19. Additionally, it would be of interest to evaluate if individuals subclinically infected with COVID-19 with no external clinical signs would have ferritin in normal values, as described in other infectious diseases [45]. Second, the study did not consider the compounding effects of gender, age, or nutritional status. Third, no objective criteria such as hemoglobin or transferrin levels in saliva were employed to identify the presence of blood in saliva. However, no individuals showed apparent oral disease or salivary blood contamination detected by visual inspection. In addition, the lower limit of quantification of the assay was 4.1 µg/L, and therefore, it did not show values below this point for some healthy individuals. Lastly, it should be borne in mind that ferritin is a major acute-phase protein in humans, and its increases are not specific to COVID-19. 

## 5. Conclusions

Ferritin in saliva can be measured rapidly, economically, and in a reproducible way. This assay was not affected significantly by common SARS-CoV-2 inactivation methods, including 65 and 92 °C heating, NP-40, and Triton X-100, except for the addition of 0.5% SDS. Salivary ferritin levels were higher in COVID-19 patients, and the highest values were found in those patients presenting severe symptomatology. Therefore, ferritin in saliva has the potential to be a biomarker of use to evaluate severity in patients with COVID-19.

## Figures and Tables

**Table 1:** Ferritin in saliva after the different inactivation SARS-CoV-2 treatments. Results were normalized according to NT measurements, which was considered 100% and expressed as median (25–75 percentile). Asterisk indicates differences of statistical relevance vs. NT measurements (*: *p* < 0.05). NT: no inactivation treatment (control); H65: 65 °C heat; H92: 92 °C heat; SDS: 0.5% SDS; NP: 0.5% NP-40; TX100: 0.5% Triton X-100.

	NT	H65	H92	SDS	NP	TX100
Ferritin (%)	100	101 (98.4–114)	91.9 (90–105)	111 (111–121) *	107 (106–115)	115 (107–117)
